# Health insurance coverage, healthcare use, and financial protection amongst people with disabilities in Indonesia: analysis of the 2021 National Socioeconomic Survey

**DOI:** 10.1016/j.lansea.2025.100631

**Published:** 2025-07-09

**Authors:** Luthfi Azizatunnisa’, Ari Probandari, Hannah Kuper, Lena Morgon Banks

**Affiliations:** a International Centre for Evidence in Disability, London School of Hygiene and Tropical Medicine, London, UK; b Department of Health Behaviour, Environment and Social Medicine, Faculty of Medicine, Public Health and Nursing, Universitas Gadjah Mada, Yogyakarta, Indonesia; c Department of Public Health, Faculty of Medicine, Universitas Sebelas Maret, Surakarta, Indonesia

**Keywords:** Health financing, Universal health coverage, Health equity, Disability, Indonesia

## Abstract

**Background:**

*Jaminan Kesehatan Nasional* (JKN), Indonesia’s mandatory national health insurance scheme and the world’s largest single-payer system, has not been rigorously evaluated for its reach and effectiveness amongst people with disabilities, who often have greater healthcare needs. This study evaluates JKN coverage and its association with healthcare use and financial protection for people with disabilities in Indonesia.

**Methods:**

This cross-sectional study analysed the Indonesia National Socioeconomic Survey (Susenas) March 2021 dataset (n = 1,277,497). Disability was measured using the Washington Group Short Set (WG-SS). We used multivariate logistic regression to examine associations between disability and health insurance coverage, and between insurance coverage and healthcare utilization, out-of-pocket payments (OOP), and catastrophic health expenditure (CHE).

**Findings:**

Around 30% of people with disabilities were uninsured, and 35% were not enrolled in JKN, with coverage lower in the lowest socioeconomic groups, living in rural areas, or self-employment. Among JKN-enrolees, they were more likely to be in the subsidised group (vs. contributory) compared to those without disabilities. Overall, people with disabilities utilised healthcare services more frequently and incurred higher OOP and CHE than those without disabilities. These disparities were not mitigated by insurance coverage. Indeed, people with disabilities, even with JKN coverage, were more likely to experience high OOP and CHE, with those in the contributory group facing a higher likelihood of CHE than the subsidised group.

**Interpretation:**

There are large gaps in health insurance coverage for people with disabilities in Indonesia. Additionally, there is an urgent need to enhance the financial protection of people with disabilities, ensuring equitable and comprehensive care.

**Funding:**

This study is part of the first author’s PhD project, funded by the 10.13039/501100014538Indonesia Endowment Fund for Education (LPDP).

**Research in context**

**Evidence before this study**

We conducted a systematic review assessing health insurance coverage among people with disabilities, and the association between health insurance, in turn, and healthcare use, financial protection, and health status for this population in low- and middle-income countries (LMICs). We searched nine databases (Medline, Embase, Cochrane, CINAHL, EconLit, Web of Science, Global Health, Scopus, and PsyInfo) using the search strategy concept of ‘people with disabilities’ AND ‘health insurance’ AND ‘low and middle-income countries’ on Jan 25, 2023. We identified 38 relevant articles. Notably, one-third of these studies were conducted in China, and nearly half were from the WHO West Pacific region. However, there was a striking lack of research from Southeast Asia and the Western Mediterranean, and no studies from Indonesia. This is an important evidence gap considering Indonesia’s status as the country with the largest single-payer health insurance system in the world. Most of the studies (68%) focused on assessing health insurance coverage among people with disabilities, which was typically low. Almost half of the studies (45%) examined the relationship between health insurance and healthcare use, and showed a positive association with the use of disability-related services, but very limited and inconsistent research on general healthcare use. Few studies (n = 6) explored the association between health insurance and financial protection, with inconclusive findings.

**Added value of this study**

This study addresses the gaps identified in the systematic review by providing evidence from Indonesia. It examines health insurance coverage and its association with healthcare use and financial protection among people with disabilities for the world’s largest single-payer health insurance system. First, a large proportion of people with disabilities remain not enrolled in the *Jaminan Kesehatan Nasional* (JKN) (35%) and lack any form of insurance coverage (30%). Second, living in rural areas, being self-employed, and poverty are factors associated with non-enrolment in JKN for people with disabilities. Third, JKN appears to promote healthcare utilisation among people with disabilities. Still, people with disabilities face higher odds of out-of-pocket payments (OOP), and catastrophic health expenditure (CHE) even if covered by JKN compared to those without insurance. Those without insurance are less likely to access care, which might indicate unmet health needs. Fourth, among people with disabilities enrolled in JKN, those in the subsidised group, which often is people from poorer socioeconomic status, use healthcare less frequently than the contributory group, indicating other barriers beyond healthcare costs that require policy attention.

**Implications of all the available evidence**

The study’s findings highlight the need for targeted strategies to improve JKN enrolment among people with disabilities and across different types of functional difficulties. Furthermore, the programme should ensure that the eligibility criteria for subsidised enrolment address the needs of people with disabilities across all socioeconomic groups. There is also a need to expand the JKN benefits package to include essential services to close care gaps that lead to high OOP and CHE. Efforts should also focus on improving access and quality care within the JKN health facilities network to encourage service utilisation and prevent CHE.

## Introduction

There are estimated 1.3 billion people with disabilities in the world—16% of the global population.^1^^,^^2^ In Indonesia, the National Team for the Acceleration of Poverty Reduction (*Tim Nasional Percepatan Penanggulangan Kemiskinan*—TNP2K), using the National Socioeconomic Survey 2023 March, reported 2% prevalence of people with disabilities in Indonesia, measured using the Washington Group Short-Set (WG-SS) with the cut-off of a lot of difficulties and cannot do at all.^3^

People with disabilities, on average, have higher health needs than those without disability because they experience greater general healthcare needs, health needs related to their impairment (e.g., specialist care, rehabilitation, assistive technology), and health needs related to secondary health conditions.^4^ Despite their greater need for healthcare, people with disabilities frequently face greater barriers to accessing healthcare. Financial barriers are particularly important since people with disabilities are more likely to live in poverty than those without disabilities,^5^, ^6^, ^7^, ^8^ yet often incur higher healthcare costs, including direct (e.g., fees for services, medical products), indirect (e.g., transportation) and opportunity costs (e.g., their/caregiver's time spent accessing care).^9^, ^10^, ^11^, ^12^, ^13^ Therefore, they often have high out-of-pocket expenditures or forego needed health services,^11^^,^^14^ which can affect their functioning and well-being and push them further into poverty.^5^^,^^8^^,^^12^^,^^15^, ^16^, ^17^ This pattern is also seen in people with disabilities in Indonesia, as they face significant extra costs of living, including health, increasing their risk of poverty and financial precarity.^18^, ^19^, ^20^

Health insurance is a mechanism to protect against impoverishing and catastrophic health expenditures.^21^ Studies amongst general population across low- and middle-income countries (LMICs) have found that health insurance is associated with improved access to healthcare, financial protection, and health status.^22^ Yet in some settings, people with disabilities are less likely to have health insurance, as lower participation in formal employment and higher levels of poverty that can prevent access to available schemes.^23^^,^^24^ Additionally, people with disabilities may not experience the same levels of impact from health insurance as the general population. For example, health insurance may not cover the necessary care, such as rehabilitation and assistive technology (AT), reducing financial protection.^25^, ^26^, ^27^ A recent systematic review revealed a lack of data and inconclusive findings of the association between health insurance and financial protection, and general healthcare utilisation amongst people with disabilities in LMICs.^28^ It did find suggestive evidence that health insurance was linked to increased access to disability-related healthcare use, such as rehabilitation, therapy, examination, and diagnosis that were related to impairments, although more studies are needed on this topic.^28^

The Indonesian government has implemented a National Health Insurance scheme—*Jaminan Kesehatan Nasional* (JKN), since 2014. Enrolment is mandatory for all residents, including foreigners living or working in Indonesia for at least six months. Membership is divided into subsidised including national subsidy (*Penerima Bantuan Iuran*—PBI) and local government subsidy, where the government pays the premium, and contributory, where employers and/or individuals pay premiums. There is no difference in the benefits package between those in subsidised and contributory groups. The Ministry of Social Affairs determines the eligibility for the subsidised group (PBI) based on poverty criteria. Eligibility for local governments subsidy is determined by each local government.^29^^,^^30^

JKN, managed by *Badan Penyelenggara Jaminan Sosial Kesehatan* (*BPJS Kesehatan* [Social Security Agency for Health]), provides basic healthcare services, including preventive, curative, and rehabilitative care. It covers medical services (e.g., care, medicine, devices) and non-medical services like hospital rooms and ambulances. Studies amongst the general population and amongst those in lower socioeconomic and rural areas have shown that JKN improves healthcare access and offers some financial protection.^31^^,^^32^ However, notable gaps remain for people with disabilities, for example, JKN covers only a limited range of assistive products (i.e., eyeglasses, hearing aids, dental prostheses, spinal corsets, neck collars, crutches, and mobility prostheses), and financial protection remains low.^33^ Rehabilitation coverage also remains limited.^3^

In 2024, the JKN covers 277.5 million people (98% of the total population), making JKN the biggest single-payer health insurance scheme in the world.^34^^,^^35^ However, there is a lack of evidence on coverage amongst people with disabilities and its association with financial protection, and healthcare utilisation in this population. Understanding this relationship is crucial for monitoring and evaluating JKN’s effectiveness, and we cannot leave people with disabilities behind in achieving Universal Health Coverage (UHC). Therefore, this study aims to assess the coverage of JKN among people with disabilities, identify the predictors of its enrolment, and assess its association with health care utilisation and financial protection.

## Methods

### Study design, data source, and study participants

This cross-sectional study used datasets from the Indonesia National Socioeconomic Survey—*Survei Sosial Ekonomi Nasional*, collected in March 2021 (Susenas March 2021). Susenas, organized by the Central Bureau of Statistics of Indonesia (*Badan Pusat Statistik*), collects data twice yearly, in March and September.^36^ We used data from the survey’s core module (including variables on demographics, functional difficulties, healthcare utilization, and health insurance) and expenditure module (including variables on food and non-food expenditure).

The Susenas March 2021 data were collected from 345,000 households across 514 districts or cities in 34 provinces in Indonesia. However, households in special census blocks, such as boarding houses and prisons, were excluded. In the core module, household data (e.g., address, size) was reported by the household head, while individual data (e.g., education, marital status, employment, functional difficulties, health insurance, healthcare use) were self-reported by the individuals. In the expenditure module, the household head reported household expenses (e.g., rent, electricity, healthcare), while individuals reported their own consumption items (e.g., food, cigarettes). For those who could not answer the individual questions directly, the questions were asked to the head of household or the spouse.^36^ The final sample consisted of 340,032 households (1,277,497 individuals), which is nationally representative of Indonesia’s population of 271.6 million people. With this large sample size, the estimations can be made down to the district level.^37^

This study obtained ethical approval from Research Ethics Committee of London School of Hygiene and Tropical Medicine (Ref: 30136), and Faculty of Medicine, Public Health and Nursing, Universitas Gadjah Mada, Indonesia (Ref: KE/FK/0410/EC/2024).

### Procedures

#### Outcome variables

The outcome variables were health insurance coverage, healthcare utilization, out-of-pocket payment (OOP), and catastrophic health expenditure (CHE).

Health insurance coverage refers to the proportion of individuals who were enrolled in a health insurance scheme. Health insurance status was assessed using the question, “*What health insurance does (name) have?*” in which the responses were categorized into JKN (for those who had JKN coverage only), multiple (for those had JKN and other insurance), other health insurance (those who only had other insurance i.e., *Jamkesda*/local insurance, private, company), and no insurance.

Healthcare utilization was measured based on participants’ reported use of outpatient and inpatient services. For outpatient care, participants indicated whether they had used such services, and how many times they had accessed outpatient services in the past month. For inpatient care, participants self-reported whether they had been hospitalized in the past year, and the duration of their stay (in days).

Out-of-pocket (OOP) payment refers to direct payments for healthcare-related expenses, including doctor’s fees, medication or assistive technology (AT) purchases, and hospital bills, excluding health-related transportation and special nutrition costs. OOP payments were categorised into tertiles, representing low, middle and high expenditures group. CHE was calculated using the WHO definition, where health expenditure is deemed catastrophic if the OOP payments equal or exceed 40% of a household’s non-subsistence expenditure.^38^

#### Disability measure

Disability was measured using the Washington Group Short Set (WG-SS), which asked about functional difficulties in walking or climbing stairs, seeing, hearing, remembering or concentrating, communication, and self-care. There were two additional questions on the difficulties in moving fingers and experiencing behaviour and/or emotional disturbance. Functional difficulties questions were asked to individuals aged two years and above or their guardian. People were considered to have a disability if they reported “a lot of difficulties” or “cannot do at all” in at least one domain, as per the international recommendation, or “always” or “frequently” in behaviour or emotion domain.^39^

#### Other data collected

Demographics and socioeconomic variables such as sex, age, residence, household size, marital status, education level, work status, and wealth were included in the analysis.

Sex refers to biological sex at birth, categorised as female and male. Age was measured in years and grouped into six categories: <18, 18–30, 31–40, 41–50, 51–60, and >60. Residence refers to the household’s living area, classified as either urban or rural, according to the Central Bureau of Statistics criteria. Household size was categorised into three groups based on data distribution: 1–2 members, 3–4 members, and five or more members. Marital status was divided into three categories: never married, married, and divorced/widowed.

Education level refers to the highest level of formal education completed, categorised as follow: no schooling or less than elementary, elementary (grades 1–6), junior high school (grades 7–9), senior high school (grades 10–12), and higher education (college or university). Education data were collected from individuals aged five years and above.

Work status refers to whether an individual was engaged in any work during the past week for at least one uninterrupted hour. It was grouped into not working (e.g., students, housewives, those who were unemployed), working—self-employed (e.g., entrepreneur, freelancer, farmer), and working for others (e.g., employees). This information was collected from individuals aged ten years and above. Socioeconomic status (SES) was measured as the average monthly total expenditure per capita and grouped into quintiles.

### Statistical analysis

Statistical analysis was conducted using Stata 18. Descriptive statistics were performed to explore the datasets. Logistic regression or multinomial logistic regression was employed to analyse the following: (i) the probability of insurance enrolment comparing individuals with and without disabilities, (ii) coverage of subsidised JKN by socioeconomic group comparing people with vs. without disabilities, (iii) factors associated with health insurance enrolment amongst individuals with disabilities, (iv) healthcare use and CHE comparing individuals with and without disabilities, (v) associations between health insurance and healthcare utilization, and CHE amongst people with disabilities. Detailed information on each analysis, including variables and statistical tests, can be found in Supplementary Material 1.

The variables of disability, health insurance, outpatient, and inpatient use had no missing data, while 5% of the data for OOP payments and CHE were missing. We used a complete case approach to analyse the data. The analyses accounted for stratification and weighting factors to address sampling complexities, using the svyset and svy commands. We conducted the analysis with two models: Model 1 controlling for age and sex (i.e., adjusting for their potential influence in the tested association), while Model 2 further controlling for age, sex, household size, and urban/rural status.

Results are presented as Adjusted Odds Ratios (AORs) in the main text. Additional analyses reporting Average Marginal Effects (AMEs) in percentage points (PP) are available in Supplementary Materials 2–6.

### Role of the funding source

The study’s funding sources were not involved in the research design, data collection, analysis, interpretation, or writing of the report.

## Results

### Characteristics of the study population

This analysis estimated the prevalence of disability in the population to be 2.4%. Individuals with disabilities were more likely to be older, female, and living in rural areas (Table 1). People with disabilities were more likely to receive no formal education (48% vs. 26%), have no work (77% vs. 49%), and have the lowest expenditure per capita (27% vs. 20%) than those without disability. Among individuals with disabilities, the three most common types of functional difficulties were difficulties in walking or climbing stairs (43%), seeing (33%), and remembering/concentrating (28%).

**Table 1:** Characteristics of the study population.

Variables	Disability n = 28,451	No Disability n = 1,206,600
Sex		
Male	46%	50%
Female	54%	50%
Age group (years)		
<18	7%	28%
18–30	7%	22%
31–40	6%	16%
41–50	8%	14%
51–60	15%	11%
>60	56%	9%
Residence		
Urban	51%	57%
Rural	49%	43%
Marital status		
Never married	19%	41%
Married	46%	52%
Widowed or divorced	35%	7%
Education level		
Less than elementary	48%	26%
Elementary	27%	24%
Junior high school	10%	18%
Senior high school	11%	24%
University or college	4%	8%
Working status		
Not working	77%	49%
Working—self-employment	17%	27%
Working—for other	6%	24%
Household size		
1–2 member	33%	11%
3–4 member	37%	51%
5+ member	30%	38%
Expenditures (per capita) quintiles		
Lowest	27%	20%
Second	21%	20%
Third	18%	20%
Fourth	18%	20%
Highest	16%	20%
Experiencing catastrophic health expenditure (CHE)		
Yes	3%	1%
No	97%	99%
Functional difficulties (not mutually exclusive)		
Seeing	33%	–
Hearing	25%	–
Walking or climbing stairs	43%	–
Using fingers	17%	–
Remembering or concentrating	28%	–
Behaviour and/or emotion	13%	–
Speaking and/or communication	21%	–
Self-care	22%	–

### Probability of enrolment in health insurance amongst people with vs. without disabilities

Approximately 65% of people with disabilities were enrolled in JKN, while around 30% of people with disabilities did not have any health insurance coverage, compared to 61% and 31% among those without disabilities (Table 2). No significant difference in odds of JKN only enrolment (vs. no insurance) was observed between the two groups. Further, AME shows that the probability of people with disability to have JKN coverage was 1.11 percentage points higher than those without disabilities (Supplementary 2). People with disabilities were less likely to have multiple (AOR 0.88 [0.79–0.97]) or other health insurance (AOR 0.80 [0.73–0.87]) compared to those without disabilities. Functional difficulties in hearing, remembering or concentrating, controlling behaviour or emotion, and communication were slightly less likely to have JKN (vs. no insurance) than those without disabilities.

**Table 2:** Probability of enrolment in health insurance of people with vs. without disabilities and across functional difficulties.

Functional difficulties	Insurance status	Percentage		Disability vs. non-disability	
		Disability n = 28,451	Non-disability n = 1,206,600	Model 1 AOR (95% CI)	Model 2 AOR (95% CI)
All disability	Jaminan Kesehatan Nasional (JKN)	61%	57%	Baseline	Baseline
	Multiple (JKN + other insurance)	4%	4%	0.87 (0.78–0.97)^b^	0.88 (0.79–0.97)^a^
	Other insurance	5%	8%	0.81 (0.74–0.88)^b^	0.80 (0.73–0.87)^b^
	No insurance	30%	31%	1.04 (0.99–1.09)	1.01 (0.96–1.05)
	**Among JKN enrolees** Subsidised (vs. contributory)	75%	64%	1.50 (1.41–1.59)^b^	1.46 (1.38–1.55)^b^
Seeing	JKN	60%	57%	Baseline	Baseline
	Multiple (JKN + other insurance)	3%	4%	0.84 (0.70–1.01)	0.85 (0.71–1.02)
	Other insurance	6%	8%	0.95 (0.81–1.11)	0.94 (0.80–1.09)
	No insurance	30%	31%	1.10 (1.02–1.19)^a^	1.04 (0.97–1.13)
	**Among JKN enrolees** Subsidised (vs. contributory)	76%	64%	1.59 (1.43–1.76)^b^	1.47 (1.33–1.64)^b^
Hearing	JKN	58%	57%	Baseline	Baseline
	Multiple (JKN + other insurance)	3%	4%	0.81 (0.66–1.01)	0.83 (0.66–1.03)
	Other insurance	6%	8%	0.91 (0.76–1.07)	0.89 (0.75–1.06)
	No insurance	33%	31%	1.22 (1.12–1.33)^b^	1.16 (1.06–1.26)^b^
	**Among JKN enrolees** Subsidised (vs. contributory)	78%	64%	1.71 (1.50–1.94)^b^	1.62 (1.42–1.84)^b^
Walking/ climbing stairs	JKN	63%	57%	Baseline	Baseline
	Multiple (JKN + other insurance)	4%	4%	0.94 (0.82–1.09)	0.95 (0.83–1.09)
	Other insurance	5%	8%	0.74 (0.65–0.85)^b^	0.74 (0.65–0.84)^b^
	No insurance	28%	31%	0.98 (0.91–1.05)	0.96 (0.89–1.03)
	**Among JKN enrolees** Subsidised (vs. contributory)	72%	64%	1.24 (1.15–1.35)^b^	1.23 (1.14–1.34)^b^
Using fingers	JKN	61%	57%	Baseline	Baseline
	Multiple (JKN + other insurance)	3%	4%	0.83 (0.67–1.04)	0.83 (0.67–1.04)
	Other insurance	5%	8%	0.68 (0.55–0.84)^b^	0.68 (0.55–0.84)^b^
	No insurance	31%	31%	1.07 (0.96–1.19)	1.06 (0.95–1.18)
	**Among JKN enrolees** Subsidised (vs. contributory)	73%	64%	1.33 (0.14–0.41)^b^	1.32 (1.15–1.51)^b^
Remembering/ concentrating	JKN	58%	57%	Baseline	Baseline
	JKN + other insurance	3%	4%	0.76 (0.64–0.91)^b^	0.77 (0.64–0.92)^b^
	Other insurance	6%	8%	0.86 (0.74–1.01)	0.86 (0.74–1.00)
	No insurance	33%	31%	1.21 (1.12–1.31)^b^	1.18 (1.09–1.28)^b^
	**Among JKN enrolees** Subsidised (vs. contributory)	78%	64%	1.77 (1.59–1.98)^b^	1.76 (1.57–1.98)^b^
Behaviour/ emotion	JKN	59%	57%	Baseline	Baseline
	Multiple (JKN + other insurance)	3%	4%	0.76 (0.59–0.99)^a^	0.77 (0.59–1.00)
	Other insurance	6%	8%	0.77 (0.62–0.95)^a^	0.76 (0.62–0.94)^a^
	No insurance	33%	31%	1.13 (1.00–1.27)^a^	1.10 (0.98–1.24)
	**Among JKN enrolees** Subsidised (vs. contributory)	77%	64%	1.81 (1.55–2.12)^b^	1.81 (1.53–2.13)^b^
Communication	JKN	57%	57%	Baseline	Baseline
	Multiple (JKN + other insurance)	4%	4%	0.91 (0.75–1.12)	0.92 (0.75–1.13)
	Other insurance	6%	8%	0.88 (0.74–1.04)	0.87 (0.73–1.03)
	No insurance	33%	31%	1.17 (1.08–1.28)	1.14 (1.04–1.25)^b^
	**Among JKN enrolees** Subsidised (vs. contributory)	76%	64%	1.70 (1.50–1.92)^b^	1.66 (1.46–1.89)^b^
Self-care	JKN	59%	57%	Baseline	Baseline
	Multiple (JKN + other insurance)	4%	4%	1.08 (0.89–1.31)	1.09 (0.89–1.32)
	Other insurance	6%	8%	0.91 (0.77–1.08)	0.91 (0.77–1.08)
	No insurance	31%	31%	1.08 (0.99–1.18)	1.09 (0.99–1.19)
	**Among JKN enrolees** Subsidised (vs. contributory)	70%	64%	1.16 (1.05–1.30)^b^	1.18 (1.05–1.32)^b^

AOR: Adjusted Odds Ratio.

Model 1: Controlling for age group, and sex.

Model 2: Controlling for age group, sex, urban/rural, and household size.

a P value < 0.05.

b P value < 0.01.

Individuals with disabilities were more likely to be enrolled in the JKN subsidised group (vs. contributory group) compared to those without disability (AOR 1.46 [1.38–1.55]). This finding was similar in all different groups of functional difficulties. People with disabilities at the lowest, fourth, and highest SES had higher odds of being in the subsidised (vs. contributory) group compared to those without disability in Models 1 and 2 (Table 3) while this pattern was observed in the second and third SES quintiles, but only in Model 1.

**Table 3:** JKN subsidised coverage in people with vs. without disabilities by socioeconomic status.

Expenditure quintile	Insurance status	Disability n = 28,451	Non-disability n = 1,206,600	Model 1 AOR (95% CI)	Model 2 AOR (95% CI)
Lowest	JKN–contributory	4%	6%	Ref	Ref
	JKN–subsidised	55%	50%	1.34 (1.12–1.62)^b^	1.32 (1.09–1.59)^b^
	Other insurance	5%	6%	1.29 (1.02–1.63)^a^	1.27 (1.00–1.61)^a^
	No insurance	36%	39%	1.36 (1.12–1.65)^b^	1.30 (1.07–1.59)^b^
Second	JKN–contributory	9%	11%	Ref	Ref
	JKN–subsidised	54%	47%	1.17 (1.00–1.36)^a^	1.11 (0.95–1.30)
	Other insurance	5%	7%	0.98 (0.76–1.25)	0.93 (0.73–1.19)
	No insurance	32%	35%	1.08 (0.92–1.26)	1.01 (0.86–1.18)
Third	JKN–contributory	15%	18%	Ref	Ref
	JKN–subsidised	52%	43%	1.19 (1.03–1.38)^a^	1.15 (0.99–1.32)
	Other insurance	5%	7%	0.73 (0.58–0.92)^b^	0.70 (0.56–0.89)^b^
	No insurance	29%	32%	1.03 (0.88–1.20)	0.97 (0.83–1.13)
Fourth	JKN–contributory	21%	28%	Ref	Ref
	JKN–subsidised	45%	35%	1.42 (1.26–1.60)^b^	1.35 (1.19–1.53)^b^
	Other insurance	5%	9%	0.89 (0.72–1.11)	0.86 (0.69–1.07)
	No insurance	29%	28%	1.37 (1.20–1.58)^b^	1.27 (1.10–1.47)^b^
Highest	JKN–contributory	43%	48%	Ref	Ref
	JKN–subsidised	31%	21%	1.43 (1.26–1.61)^b^	1.34 (1.18–1.52)^b^
	Other insurance	7%	10%	1.03 (0.82–1.30)	1.00 (0.79–1.26)
	No insurance	19%	21%	1.21 (1.05–1.39)^b^	1.11 (0.96–1.28)

AOR: Adjusted Odds Ratio; JKN: Jaminan Kesehatan Nasional.

Model 1: controlling for age group, and sex.

Model 2: controlling for age group, sex, urban/rural, and household size.

a P value < 0.05.

b P value < 0.01.

Interactions between age and sex were observed. Females aged 18–30, 31–40, 41–50, and 51–60 years had slightly higher odds of JKN-only enrolment than males of the same group (Supplementary 7). Three-way interactions between disability, age, and sex showed varying patterns. Notably, females with disabilities aged 41–50 years had significantly lower odds of having multiple or other insurance (AOR 0.50 [0.28–0.87]) compared to males without disabilities aged 0–17 years. No other significant three-way interactions were found.

### Predictors of being enrolled in any insurance among people with disabilities

Amongst people with disabilities, enrolment in JKN (vs. no insurance) was more frequent in adults, urban residents, people from larger households, those with education, married people, and people with higher SES (Table 4). Average Marginal Effects (AME) analysis saw a similar pattern (Supplementary 4).

**Table 4:** Factors associated with health insurance enrolment amongst people with disabilities.

Variables	JKN vs. no insurance		JKN vs. multiple/other insurance		**JKN** subsidised vs. contributory	
	Model 1 AOR (95% CI)	Model 2 AOR (95% CI)	Model 1 AOR (95% CI)	Model 2 AOR (95% CI)	Model 1 AOR (95% CI)	Model 2 AOR (95% CI)
Sex						
Male	Ref	Ref	Ref	Ref	Ref	Ref
Female	0.96 (0.89–1.03)	0.97 (0.91–1.05)	0.88 (0.79–0.99)^a^	0.88 (0.79–0.99)^a^	1.06 (0.97–1.16)	1.03 (0.94–1.13)
Age						
<18	Ref	Ref	Ref	Ref	Ref	Ref
18–30	1.62 (1.30–2.00)^b^	1.68 (1.35–2.09)^b^	1.50 (1.10–2.04)^a^	1.51 (1.11–2.06)^b^	1.63 (1.25–2.13)^b^	1.65 (1.25–2.18)^b^
31–40	1.38 (1.11–1.71)^b^	1.46 (1.17–1.82)^b^	1.45 (1.07–1.96)^a^	1.47 (1.08–1.99)^a^	1.50 (1.14–2.00)^b^	1.48 (1.12–1.97)^b^
41–50	1.57 (1.28–1.93)^b^	1.71 (1.39–2.10)^b^	1.72 (1.31–2.26)^b^	1.75 (1.33–2.30)^b^	1.77 (1.38–2.27)^b^	1.72 (1.34–2.23)^b^
51–60	1.96 (1.61–2.31)^b^	2.20 (1.83–2.66)^b^	2.01 (1.54–2.61)^b^	2.06 (1.58–2.69)^b^	1.37 (1.10–1.70)^b^	1.34 (1.06–1.68)^a^
>60	1.49 (1.27–1.76)^b^	1.72 (1.46–2.03)^b^	1.78 (1.42–2.24)^b^	1.86 (1.47–2.34)^b^	1.62 (1.33–1.97)^b^	1.60 (1.29–1.97)^b^
Residence						
Urban	Ref	Ref	Ref	Ref	Ref	Ref
Rural	0.64 (0.58–0.70)^b^	0.64 (0.58–0.70)^b^	0.99 (0.86–1.14)	0.99 (0.86–1.14)	2.83 (2.53–3.17)^b^	2.82 (2.52–3.16)^b^
Household size						
1–2 members	Ref	Ref	Ref	Ref	Ref	Ref
3–4 members	1.35 (1.21–1.50)^b^	1.33 (1.19–1.48)^b^	1.14 (0.96–1.34)	1.14 (0.96–1.34)	0.89 (0.77–1.02)	0.91 (0.79–1.05)
5+ members	1.33 (1.19–1.49)^b^	1.32 (1.18–1.48)^b^	1.08 (0.90–1.28)	1.08 (0.90–1.28)	0.97 (0.84–1.13)	1.00 (0.85–1.14)
Education						
No school/less than elementary	Ref	Ref	Ref	Ref	Ref	Ref
Elementary	1.31 (1.18–1.45)^b^	1.26 (1.14–1.40)^b^	1.06 (0.90–1.25)	1.05 (0.89–1.25)	0.48 (0.41–0.55)^b^	0.51 (0.44–0.58)^b^
Junior high school	1.51 (1.30–1.76)^b^	1.39 (1.19–1.62)^b^	1.28 (1.02–1.61)^a^	1.27 (1.01–1.60)^a^	0.25 (0.21–0.30)^b^	0.28 (0.24–0.34)^b^
Senior high school	2.18 (1.85–2.57)^b^	1.91 (1.62–2.27)^b^	0.83 (0.68–1.02)	0.82 (0.66–1.01)	0.10 (0.08–0.12)^b^	0.12 (0.10–0.14)^b^
College/university	2.57 (2.05–3.27)^b^	2.29 (1.80–2.92)^b^	0.77 (0.55–1.05)	0.76 (0.55–1.04)	0.08 (0.06–0.10)^b^	0.09 (0.07–0.11)^b^
Marital						
Never married	Ref	Ref	Ref	Ref	Ref	Ref
Married	1.41 (1.19–1.68)^b^	1.44 (1.20–1.72)^b^	0.95 (0.74–1.21)	0.94 (0.73–1.20)	0.48 (0.38–0.61)^b^	0.44 (0.35–0.55)^b^
Widowed/divorced	1.01 (0.83–1.21)	1.01 (0.83–1.22)	0.85 (0.64–1.12)	0.84 (0.64–1.11)	0.66 (0.51–0.85)^b^	0.61 (0.47–0.79)^b^
Working status						
Not working	Ref	Ref	Ref	Ref	Ref	Ref
Working—self-employed	0.82 (0.74–0.91)^b^	0.90 (0.81–1.01)	0.94 (0.79–1.12)	0.95 (0.80–1.13)	1.46 (1.26–1.70)^b^	1.26 (1.08–1.48)^b^
Working for other	1.23 (1.00–1.52)^a^	1.23 (0.99–1.51)	0.67 (0.52–0.88)^b^	0.67 (0.52–0.88)^b^	0.56 (0.45–0.69)^b^	0.56 (0.45–0.70)^b^
Expenditure per capita quintiles						
Lowest	Ref	Ref	Ref	Ref	Ref	Ref
Second	1.21 (1.06–1.37)^b^	1.22 (1.07–1.39)^b^	1.01 (0.83–1.24)	1.01 (0.82–1.23)	0.41 (0.33–0.52)^b^	0.42 (0.33–0.53)^b^
Third	1.40 (1.23–1.60)^b^	1.42 (1.24–1.62)^b^	1.19 (0.97–1.46)	1.18 (0.96–1.45)	0.25 (0.20–0.31)^b^	0.25 (0.20–0.32)^b^
Fourth	1.39 (1.21–1.59)^b^	1.43 (1.23–1.65)^b^	1.06 (0.86–1.30)	1.05 (0.85–1.29)	0.15 (0.12–0.19)^b^	0.14 (0.11–0.18)^b^
Highest	2.23 (1.91–2.61)^b^	2.20 (1.86–2.59)^b^	0.71 (0.57–0.87)^b^	0.69 (0.55–0.87)^b^	0.05 (0.04–0.06)^b^	0.05 (0.04–0.06)^b^

AOR: Adjusted Odds Ratio; JKN: Jaminan Kesehatan Nasional.

Model 1: controlling for age and sex; Model 2: controlling for age, sex, urban/rural, and household size.

a P value < 0.05.

b P value < 0.01.

Enrolment in JKN (vs. multiple or other insurance) was more common amongst males, adults, and individuals with junior high school education, and less common amongst those working in the formal sector and those in the highest SES group. Otherwise, there were no clear differences. Among JKN enrolees, being subsidised (vs. contributory) was more frequent amongst adults, rural residents, individuals without formal education, those who had never married, the self-employed, and those in the lowest SES.

### Healthcare utilization and catastrophic health expenditure among people with and without disabilities

People with disabilities were more likely to use both outpatient and inpatient services than those without disabilities (Table 5). They had more outpatient visits, longer hospital stays, higher OOP payments, and a greater likelihood of experiencing CHE. These patterns persisted, regardless of their health insurance status. Further analysis of average marginal effects saw a similar pattern (Supplementary 5).

**Table 5:** Healthcare utilization and financial protection of people with vs. without disabilities across insurance status in Indonesia.

	Overall disability vs. non-disability AOR (95% CI)	No insurance disability vs. non-disability AOR (95% CI)	JKN Disability vs. non-disability AOR (95% CI)	JKN-subsidised disability vs. non-disability AOR (95% CI)	JKN-contributory disability vs. non-disability AOR (95% CI)	Multiple/other insurance disability vs. non-disability AOR (95% CI)
Having outpatient visits in the last month	1.95 (1.86–2.05)^b^	1.69 (1.52–1.87)^b^	2.07 (1.95–2.19)^b^	1.88 (1.76–2.01)^b^	2.67 (2.40–2.97)^b^	2.07 (1.80–2.39)^b^
Having >1 outpatient visits^c^	1.77 (1.62–1.92)^b^	1.64 (1.36–1.96)^b^	1.80 (1.63–2.00)^b^	1.74 (1.55–1.95)^b^	1.93 (1.60–2.34)^b^	1.75 (1.33–2.30)^b^
Having inpatient visits in the last year	2.33 (2.17–2.49)^b^	1.64 (1.37–1.98)^b^	2.54 (2.35–2.75)^b^	2.35 (2.14–2.58)^b^	3.14 (2.73–3.60)^b^	2.18 (1.75–2.73)^b^
Hospital length of stay ≥3 days^c^	1.70 (1.44–2.01)^b^	1.66 (1.09–2.54)^a^	1.65 (1.35–2.01)^b^	1.54 (1.24–1.90)^b^	2.04 (1.41–2.94)^b^	1.91 (1.17–3.09)^b^
Out-of-pocket payment						
Lowest	Ref	Ref	Ref	Ref	Ref	Ref
Second	0.95 (0.90–1.00)	0.89 (0.81–0.98)^a^	0.99 (0.93–1.06)	1.04 (0.97–1.11)	1.05 (0.89–1.24)	0.97 (0.82–1.14)
Highest	1.25 (1.18–1.31)^b^	1.16 (1.05–1.29)^b^	1.33 (1.25–1.41)^b^	1.47 (1.37–1.58)^b^	1.60 (1.37–1.86)^b^	1.18 (1.00–1.38)^a^
Experiencing catastrophic health expenditure (40% threshold)	2.25 (1.98–2.56)^b^	1.83 (1.43–2.34)^b^	2.32 (2.00–2.70)^b^	2.22 (1.84–2.69)^b^	3.04 (2.39–3.86)^b^	2.79 (1.78–4.38)^b^

AOR: Adjusted Odds Ratio; controlling for age, sex, urban/rural, and household size; JKN: Jaminan Kesehatan Nasional.

a P value < 0.05.

b P value < 0.01.

c Among those using the services.

### Health insurance status, healthcare utilization, and financial protection among people with disabilities

Amongst people with disabilities, being enrolled in JKN (vs. no insurance) was positively associated with having outpatient visits (AOR 1.58 [1.42–1.77]), multiple outpatient visits (AOR 1.22 [1.02–1.47]), inpatient care (AOR 3.38 [2.83–4.08]), hospitalisation ≥3 days (AOR 1.96 [1.26–3.05]), higher OOP payment, and experiencing CHE (AOR 1.83 [1.41–2.38]) (Table 6).

**Table 6:** Association between health insurance status and healthcare utilization, and financial protection among people with disabilities in Indonesia.

Outcome variables	No insurance n: 7634	JKN n: 18,177	JKN—subsidised n: 14,946	JKN—contributory n: 4293	Multiple/other insurance n: 2640	AOR (95% CI)		
						JKN vs. no insurance	JKN vs. multiple/other insurance	JKN subsidised vs. contributory
Having outpatient visits in the last month	19%	28%	26%	35%	26%	1.58 (1.42–1.77)^c^	1.07 (0.92–1.24)	0.69 (0.61–0.78)^c^
Having >1 outpatient visits^a^	35%	40%	41%	37%	38%	1.22 (1.02–1.47)^b^	1.07 (0.82–1.39)	1.10 (0.90–1.35)
Having inpatient visits in the last year	4%	11%	10%	17%	9%	3.38 (2.82–4.07)^c^	1.30 (1.05–1.61)^b^	0.52 (0.44–0.60)^c^
Hospital length of stay ≥3 days^a^	75%	87%	84%	91%	86%	1.96 (1.26–3.05)^c^	1.02 (0.62–1.67)	0.62 (0.43–0.91)^b^
Out-of-pocket payment								
Lowest	46%	33%	38%	15%	32%	Ref	Ref	Ref
Second	29%	28%	29%	23%	29%	1.28 (1.15–1.44)^c^	0.92 (0.78–1.10)	0.51 (0.43–0.62)^c^
Highest	25%	40%	33%	62%	39%	2.03 (1.81–2.28)^c^	0.98 (0.83–1.16)	0.22 (0.19–0.27)^c^
Experiencing catastrophic health expenditure (40% threshold)	2%	3%	3%	6%	4%	1.83 (1.41–2.38)^c^	0.89 (0.57–1.40)	0.52 (0.39–0.69)^c^

AOR: Adjusted Odds Ratio–controlling for age, sex, household size, urban/rural; JKN: Jaminan Kesehatan Nasional.

a Among those using the services.

b P value < 0.05.

c P value < 0.01.

Compared to multiple/other insurance, being enrolled in JKN was only associated with higher odds of inpatient use (AOR 1.30 [1.05–1.61]). No further association observed. Opposite trends were observed among those JKN-subsidised vs. contributory. JKN subsidised group was associated with lower odds of having outpatient visits (AOR 0.69 [0.61–0.78]), inpatient care (AOR 0.52 [0.44–0.60]), hospitalisation ≥3 days (AOR 0.62 [0.43–0.91]), higher OOP payments, and experiencing CHE (AOR 0.52 [0.39–0.69]).

## Discussion

This study investigates health insurance coverage amongst people with disabilities in Indonesia and its association with healthcare utilisation and financial protection. The findings reveal that a large portion of individuals with disabilities are either uninsured (30%) or not enrolled in JKN (35%). When controlling for age, sex, residence area, and household size, there was no difference in odds of being enrolled in JKN (vs. no insurance) between people with and without disabilities. People with disabilities were more likely to be in the subsidised group (vs. contributory group). They exhibited higher healthcare utilisation, OOP payments, and higher odds of experiencing CHE. Interestingly, those enrolled in JKN experienced higher odds of CHE than individuals without insurance. Notably, amongst individuals with disabilities enrolled in JKN, those in the contributory group demonstrated higher healthcare use, greater odds of CHE, and higher OOP than the subsidised group.

There was no difference in the odds of having JKN coverage between people with and without disabilities. A similar pattern was also reported in other national health insurance schemes in Peru, and Rwanda,^27^^,^^40^, ^41^, ^42^ while lower odds among people with disabilities were observed in Brazil,^24^ and higher odds were observed in Vietnam, Colombia.^16^^,^^43^ Variations in health system structures, insurance financing mechanisms, and population characteristics likely account for these differences.^22^

The high proportion of people with disabilities who are uninsured in Indonesia highlights the need for further evaluation, particularly regarding the barriers to JKN enrolment. A recent report suggests that the absence of a citizen identification document (ID), compounded by stigma and limited access to public health services, is a significant barrier to enrolment.^44^

People with disabilities are often excluded from the formal employment market and thus have a higher likelihood of being out of work or working in the informal sector.^45^ While JKN is mandatory for all citizens, enforcing this requirement in the informal sector poses significant challenges, as individuals must register voluntarily. In contrast, there are robust monitoring mechanisms for JKN enrolment for formal sector workers, as the premiums are paid partly by the employers, and the employees’ contributions are deducted directly from their salaries. Barriers to enrolment for informal workers include irregular income, which complicates premium payments, misinformation or a lack of understanding about the system, and fluctuating healthcare needs.^46^^,^^47^

Rural residents with disabilities may face barriers to JKN enrolment, including limited healthcare access and availability, thus lowering their motivation to enrol in JKN, transportation difficulties to the JKN enrolment office, lack of information, and a high prevalence of informal workers.^48^^,^^49^ These challenges underscore gaps in JKN accessibility and equity.

JKN coverage among people with disabilities is linked to increased healthcare utilization, implying that JKN promotes healthcare access. This finding aligns with several studies conducted in Vietnam and China.^50^, ^51^, ^52^ However, improved access is also associated with higher out-of-pocket expenses and a greater odds of catastrophic health expenditures compared to those without insurance. This finding suggests that uninsured individuals may forgo necessary care, highlighting unmet healthcare needs that require further study.

Overall, even with health insurance coverage, people with disabilities are more likely to have higher OOP payments and experiencing CHE compared to people without disability. This finding can be explained by: (i) People with disabilities have higher levels of health needs, thus spending extra cost for health^51^^,^^52^; (ii) the existing insurance provides no or limited coverage for the necessary care^52^; and (iii) due to healthcare availability and accessibility issues, they may resort to using private services,^50^ hence they pay out-of-pocket.^51^ Disparities in the distribution, accessibility, and readiness of healthcare facilities, especially in eastern Indonesia, where public health centres (*Puskesmas*) ratios are below average, remain a major barriers.^53^ Supply-side constraints, particularly the lack of *Puskesmas*, and poverty significantly limit JKN’s impact on healthcare utilisation.^54^

Individuals with disabilities are more likely to be enrolled in the JKN subsidised group, particularly those with lower socioeconomic status, rural residents, and self-employed individuals, aligning with the programme’s goal of targeting those in poverty. However, those without insurance are also concentrated amongst the poor, rural, and self-employed, suggesting that JKN subsidies may need to reach those facing barriers to contributory enrolment better. Additionally, the programme should ensure that the criteria for subsidised enrolment are responsive to the needs of people with disabilities across all socioeconomic levels, not only those in the lowest SES group.

Despite having the same benefit package for both subsidised and contributory groups, people with disabilities in the contributory group face higher OOP expenses and are more likely to experience CHE. This may reflect their greater financial capacity to cover care not included in the insurance. In contrast, subsidised group members, often from poorer households, may forgo unaffordable care, non-covered services, or care with high indirect costs like transport or lost work time.^50^^,^^55^ Further research is needed to examine unmet healthcare needs among people with disabilities by insurance type and coverage.

A discrepancy exists between coverage reported by BPJS Kesehatan and this study. BPJS Kesehatan reported a coverage of 98% in October 2024,^34^ and 86% in 2021,^56^ while this analysis, using *Susenas* 2021 data, estimates 61%. This gap stems from differing data sources: *BPJS Kesehatan* uses enrolment records, while *Susenas* relies on self-reported data, which may underestimate coverage due to participants’ unawareness of their enrolment or lack of a physical JKN card.

Similarly, the finding that children are less likely to be enrolled in JKN may suggest these possibilities: (i) children in non-enrolled households may come from poor households with relatively low literacy on health insurance,^57^ or (ii) misunderstandings during data collection may have occurred. For example, since children are covered under their parents’ JKN, parents might have mistakenly reported that their children are not covered by health insurance. These findings underscore the need for improved public awareness of JKN enrolment and benefits and for *BPJS Kesehatan* to disaggregate data by disability status for more accurate evaluations.

This study highlights that people with disabilities use more healthcare services than those without disabilities, reflecting their complex and ongoing medical needs. Despite insurance coverage, individuals with disabilities are at risk of CHE, indicating the heavy financial burden of healthcare. Several policy recommendations are proposed to address these issues. First, expanding insurance coverage for individuals with disabilities, particularly targeting those who are poor, live in rural areas, are self-employed, or have invisible disabilities, is crucial. A community-based approach, such as *Dasawisma* linked to the health system, can be an effective strategy to identify individuals not enrolled in JKN and recommend them for subsidy enrolment.^58^ Second, the benefits package should be reassessed to ensure it adequately meets the essential health needs of people with disabilities, especially in terms of assistive technology (AT) and rehabilitation services, which currently have limited coverage.^59^ Additionally, efforts should also focus on enhancing the availability, readiness, and accessibility of quality care within the JKN health facilities network to encourage service utilization and reduce the risk of CHE, as these issues were also challenges in other settings.^60^ Third, people with disabilities are more likely to be in the subsidised group, and this group are less likely to use healthcare compared to those in the contributory group. As enrolment in the subsidised group is often linked to proxies of poverty (e.g., low expenditures, self-employment, low education, rural residents), financial barriers remain, and financial support to cover both direct and indirect costs is essential. Furthermore, there is a need for further research, including investigating the specific costs that lead to OOP payments, calculating their amount, and identifying the unmet healthcare needs for this population.

The strength of this study lies in its use of a large, nationally representative dataset, providing valuable insights into healthcare utilization and CHE across Indonesia. This robust sample size allows for generalisable findings and a comprehensive understanding of the issues faced by people with disabilities.

However, there are several limitations to consider. First, due to the cross-sectional nature of the dataset, the impact of health insurance on the outcomes of interest cannot be assessed. Second, the disability-related data only include individuals aged 2 years and older, which may limit the generalisability of the findings, especially regarding the healthcare needs and financial burdens of young children with disabilities. Third, as this study relies on secondary datasets, we are limited to the available variables, which may not capture all relevant factors. For example, health expenditures data does not specifically inquire about many forms of AT (only on glasses, prostheses, and wheelchairs), which may underestimate total health expenditures. Additionally, due to data limitations, we were unable to assess the unmet health needs of people with disabilities in this analysis.

This study underscores that a large proportion of people with disabilities remain uninsured despite their greater healthcare needs. Even among those with JKN, many continue to face CHE. To address these challenges, the study recommends improving health insurance enrolment for people with disabilities—especially those who are poor, live in rural areas, are self-employed—through targeted outreach and more effective identification processes. It also calls for a reassessment of the benefits package to ensure it adequately meets the essential health needs of this group, particularly expanding access to rehabilitation and AT. Strategies to enhance financial protection could include expanding covered services and products, and complementary social protection schemes to cover indirect and opportunity costs of seeking care.

## Contributors

LA, HK, and LMB conceptualised and designed the study. LA developed the protocol, analysed the data, interpreted the results, and drafted the manuscript. HK and LMB reviewed the protocol, interpreted the results, and reviewed the manuscript. AP was involved in interpreting results and reviewing the manuscript. All authors have reviewed, agreed upon, and are responsible for the final version of the manuscript.

## Data sharing statement

The Susenas datasets are available upon request from the Central Bureau of Statistics of Indonesia at https://www.bps.go.id/.

## Declaration of interests

HK is the principal investigator of grants from NIHR and FCDO (PENDA) on topics related to disability. LMB is the principal investigator of grants from AHRC and UNICEF on topics related to disability. LMB has received consultancy fees from IADB, World Bank, UNICEF and Special Olympics. LA has received a scholarship from the Indonesia Endowment Fund for Education (LPDP) for a PhD (2022–2026). LA signed a contract as a part-time research assistant for a project titled Synthesis and Translation of Research and Innovation from Polio Eradication, Center for Tropical Medicine, Faculty of Medicine, Public Health, and Nursing, Universitas Gadjah Mada, Yogyakarta, Indonesia. LA has also received a consultancy fee from the Missing Billion Initiative, and Thinker Labs, India. LA received travel scholarship from the London School of Hygiene and Tropical Medicine to attend a conference in Thailand and cover travel and accommodation of fieldwork in Indonesia. We declare no other competing interests.
