# Supplementary material for: Health insurance coverage, healthcare use, and financial protection amongst people with disabilities in Indonesia: analysis of the 2021 National Socioeconomic Survey
Source: Lancet Reg Health Southeast Asia. 2025 Jul 9;39:100631. doi: 10.1016/j.lansea.2025.100631 (PMC12274863; doi:10.1016/j.lansea.2025.100631)
Supplement: Supplementary 1–7 [file mmc1.docx]

**Supplementary 1.** Statistical analysis

| **Table** | **Analysis** | **Outcome variable** | **Exposure** | **Statistical test** |
| --- | --- | --- | --- | --- |
| 2 | Probability of enrolment in health insurance of people with versus without disabilities and across functional difficulties | Insurance status  1: JKN  2: Multiple (JKN + other insurance)  3: Other insurance  4: No insurance | Disability status  1: Disability  0: Non-disability  Functional difficulties vs non-disabilities | Multinomial logistic regression |
|  |  | JKN – subsidized among JKN enrolees  1: Subsidized  0: Contributory | Disability status  1: Disability  0: Non-disability  Functional difficulties vs non-disabilities | Logistic regression |
| 3 | JKN subsidised coverage between people with versus without disabilities by socioeconomic status | Insurance status  1: JKN – contributory  2: JKN – subsidised  3: Other insurance only  4: No insurance | Disability status  1: Disability  0: Non-disability | Multinomial logistic regression |
| 4 | Factors associated with health insurance enrolment among people with disabilities | Insurance status (3 categories)  1: JKN  2: Multiple & other insurance  3: No insurance  Comparison:  JKN vs no insurance  JKN vs other & multiple | Sex  Age group  Residence  Household size  Education  Employment  Marital status  Expenditure quintile | Multinomial logistic regression  Since variable insurance status has more than 2 categories, the base category was changed based on the comparison |
|  |  | JKN – subsidised among JKN enrollees  1: Subsidised  0: Contributory  Comparison: JKN subsidised vs contributory | Sex  Age group  Residence  Household size  Education  Employment  Marital status  Expenditure quintile | Logistic regression |
| 5 | Healthcare utilization and financial protection of people with versus without disabilities across insurance status | Outpatient visits in the past month (1: yes/0: no)  Outpatient visits >1 (1: yes/0: no)  Inpatient use in the past year (1: yes/0: no)  Inpatient ≥ 3 days (1: yes/0: no)  CHE (1: yes/0:no) | Disability status  1: Disability  0: Non-disability | Logistic regression |
|  |  | OOP level  1: lowest  2: second  3: highest | Disability status  1: Disability  0: Non-disability | Multinomial logistic regression |
| 6 | Association between health insurance and healthcare utilization and financial protection amongst people with disabilities | Outpatient visits (1: yes/0:no)  Outpatient visits >1 (1: yes/0:no)  Inpatient use (1: yes/0:no)  Inpatient ≥ 3 days (1: yes/0:no)  CHE (1: yes/0:no) | - JKN vs no insurance - JKN vs other & multiple - Amongst JKN enrolees: Subsidised vs contributory | Logistic regression |
|  |  | OOP level  1: lowest  2: second  3: highest | - JKN vs no insurance - JKN vs other & multiple - Amongst JKN enrolees: Subsidised vs contributory | Multinomial logistic regression |

**Supplementary 2.** Probability of Enrolment in Health Insurance of People with Versus without Disabilities and Across Functional Difficulties – Average Marginal Effects

| **Functional difficulties** | **Insurance status** | **Percentage** | | **Model 1** | **Model 2** |
| --- | --- | --- | --- | --- | --- |
|  |  | **Disability**  **n= 28,451** | **No disability**  **n= 1,206,600** | **AME – PP (95% CI)** | **AME – PP (95% CI)** |
| All disability | JKN | 61% | 57% | 0·46 (-0·57 – 1·49) | 1·11 (0·09 – 2·13)* |
|  | Multiple (JKN + other insurance) | 4% | 4% | -0·50 (-0·85 – -0·14)** | -0·43 (-0·78 - -0·07)* |
|  | Other insurance | 5% | 8% | -1·47 (-2·00 – -0·94)** | -1·46 (-2·00 – -0·93)** |
|  | No insurance | 30% | 31% | 1·50 (0·50 – 2·50)** | 0·78 (-0·22 – 1·77) |
|  | **Among JKN enrolees**  Subsidised (vs· contributory) | 75% | 64% | 8·73 (7·57 – 9·90)** | 7·72 (6·55 – 8·88)** |
| Seeing | JKN | 60% | 57% | -1·13 (-2·82 – 0·57) | -0·12 (-1·81 – 1·57) |
|  | Multiple (JKN + other insurance) | 3% | 4% | -0·73 (-1·31 – -0·14)* | -0·60 (-1·21 – 0·00) |
|  | Other insurance | 6% | 8% | -0·55 (-1·58 – 0·48) | -0·53 (-1·56 – 0·51) |
|  | No insurance | 30% | 31% | 2·41(0·75 – 4·07)** | 1·25 (-0·38 – 2·88) |
|  | **Among JKN enrolees**  Subsidised (vs· contributory) | 76% | 64% | 9·87 (7·89 – 11·87)** | 7·87 (5·85 – 9·89)** |
| Hearing | JKN | 58% | 57% | -2·86 (-4·80 - -0·93)** | -1·80 (-3·72 – 0·18) |
|  | Multiple (JKN + other insurance) | 3% | 4% | -0·91 (-1·57 - -0·26)** | -0·80 (-1·48 - -0·12)* |
|  | Other insurance | 6% | 8% | -1·11 (-2·15 – -0·06)* | -1·07 (-2·13 - -0·03)* |
|  | No insurance | 33% | 31% | 4·88 (2·98 – 6·79)** | 3·67 (1·80 – 5·55)** |
|  | **Among JKN enrolees**  Subsidised (vs· contributory) | 78% | 64% | 11·25 (8·86 – 13·64)** | 9·59 (7·20 – 11·98)** |
| Walking/ climbing stairs | JKN | 63% | 57% | 1·72 (0·22 – 3·23)* | 2·05 (0·55 – 3·55)** |
|  | Multiple (JKN + other insurance) | 4% | 4% | -0·11 (-0·63 – 0·41) | -0·07 (-0·59 – 0·45) |
|  | Other insurance | 5% | 8% | -1·85 (-2·59 – -1·11)** | -1·85 (-2·58 - -1·11)** |
|  | No insurance | 28% | 31% | 0·23 (-1·24 – 1·71) | -0·13 (-1·60 – 1·34) |
|  | **Among JKN enrolees**  Subsidised (vs· contributory) | 72% | 64% | 4·88 (3·21 – 6·56)** | 4·35 (2·70 – 5·99)** |
| Using fingers | JKN | 61% | 57% | 0·55 (-1·79 – 2·89) | 0·79 (-1·54 – 3·11) |
|  | Multiple (JKN + other insurance) | 3% | 4% | 0·55 (-1·79 – 2·89) | -0·62 (-1·35 – 0·11) |
|  | Other insurance | 5% | 8% | -2·45 (-3·53 – -1·37)** | -2·44 (-3·52 - -1·36)** |
|  | No insurance | 31% | 31% | 2·55 (0·25 – 4·85)* | 2·27 (-0·01 – 4·56) |
|  | **Among JKN enrolees**  Subsidised (vs· contributory) | 73% | 64% | 6·30 (3·60 – 8·99)** | 5·67 (2·99 – 8·34)** |
| Remembering/ concentration | JKN | 58% | 57% | -2·40 (-4·20 - -0·60)** | -1·88 (-3·66 - -0·11)* |
|  | Multiple (JKN + other insurance) | 3% | 4% | -1·09 (-1·61 – 0·57)** | -1·03 (-1·56 - -0·50)** |
|  | Other insurance | 6% | 8% | -1·36 (-2·28 – -0·43)** | -1·34 (-2·27 - -0·41)** |
|  | No insurance | 33% | 31% | 4·84 (3·09 – 6·60)** | 4·25 (2·52 – 5·97)** |
|  | **Among JKN enrolees**  Subsidised (vs· contributory) | 78% | 64% | 11·96 (9·91 – 14·00)** | 11·13 (9·07 – 13·19)** |
| Controlling behaviour/ emotion | JKN | 59% | 57% | -0·63 (-3·21 – 1·95) | -0,01 (-2·66 – 2·48) |
|  | Multiple (JKN + other insurance) | 3% | 4% | -0·99 (-1·77 – -0·21)* | -0·94 (-1·73 - -0·15)* |
|  | Other insurance | 6% | 8% | -1·88 (-3·04 – -0·72)** | -1·87 (-3·04 - -0·71)** |
|  | No insurance | 33% | 31% | 3·50 (0·96 – 6·05)** | 2·90 (0·38 – 5·43)* |
|  | **Among JKN enrolees**  Subsidised (vs· contributory) | 77% | 64% | 12·33 (9·46 – 15·20)** | 11·60 (8·71 – 14·49)** |
| Communication | JKN | 57% | 57% | -2·20 (-4·20 - -0·20)* | -1·58 (-3·56 – 0·41) |
|  | Multiple (JKN + other insurance) | 4% | 4% | -0·49 (-1·18 – 0·20) | -0·42 (-1·12 – 0·29) |
|  | Other insurance | 6% | 8% | -1·25 (-2·30 – -0·19)* | -1·23 (-2·29 - -0·18)* |
|  | No insurance | 33% | 31% | 3·94 (2·00 – 5·87)** | 3·22 (1·32 – 5·13)** |
|  | **Among JKN enrolees**  Subsidised (vs· contributory) | 76% | 64% | 11·16 (8·81 – 13·51)** | 10·13 (7·77 – 12·49)** |
| Self-care | JKN | 59% | 57% | -1·13 (-3·08 – 0·83) | -1·28 (-3·23 – 0·68) |
|  | Multiple (JKN + other insurance) | 4% | 4% | 0·25 (-0·53 – 1·03) | 0·25 (-0·53 – 1·03) |
|  | Other insurance | 6% | 8% | -0·86 (-1·95 – 0·23) | -0·85 (-1·94 – 0·25) |
|  | No insurance | 31% | 31% | 1·74(-0·10 – 3·58) | 1·88 (0·05 – 3·71)* |
|  | **Among JKN enrolees**  Subsidised (vs· contributory) | 70% | 64% | 3·43 (1·07 – 5·79)** | 3·43 (1·10 – 5·75)** |

AME: Average Marginal Effects, PP: Percentage Points

Model 1: Controlling for age group and sex

Model 2: Controlling for age group, sex, urban/rural, and household size

*P<0·05, **P<0·01

**Supplementary 3.** JKN Subsidised Coverage in People with versus without Disabilities by Socioeconomic Status – Average Marginal Effects

| **Expenditure quintile** | **Insurance status** | **Disability**  **n= 28,451** | **Non-disability**  **n= 1,206,600** | **Model 1**  **AME – PP (95% CI)** | **Model 2**  **AME – PP (95% CI)** |
| --- | --- | --- | --- | --- | --- |
| Lowest | JKN - contributory | 4% | 6% | -1·40 (-2·16 – -0·6)** | -1·34 (-2·17 – -0·51)** |
|  | JKN - subsidised | 55% | 50% | 0·63 (-1·41 – 2·67) | 1·12 (-0·93 – 3·17) |
|  | Other insurance | 5% | 6% | -0·16 (-1·03 – 0·71) | -0·10 (-0·92 – 0·72) |
|  | No insurance | 36% | 39% | 0·9 (-1·17 – 3·03) | 0·32 (-1·80 – 2·44) |
| Second | JKN - contributory | 9% | 11% | -1·05 (-2·38 – 0·29) | -0·54 (-1·95 – 0·87) |
|  | JKN - subsidised | 54% | 47% | 2·75 (0·64 – 4·85)* | 2·83 (0·72 – 4·94)** |
|  | Other insurance | 5% | 7% | -0·79 (-1·95 – 0·36) | -0·79 (-1·94 – 0·36) |
|  | No insurance | 32% | 35% | -0·91 (-2·94 – 1·11) | -1·49 (-3·49 – 0·50) |
| Third | JKN - contributory | 15% | 18% | -1·16 (-3·07 – 0·74) | -0·53 (-2·44 – 1·38) |
|  | JKN - subsidised | 52% | 43% | 4·8 (2·62 – 7·04)** | 4·76 (2·55 – 6·97)** |
|  | Other insurance | 5% | 7% | -2·39 (-3·36 – -1·42)** | -2·37 (-3·35 – -1·39)** |
|  | No insurance | 29% | 32% | -1·28 (-3·35 – 0·80) | -1·85 (-3·89 – 0·18) |
| Fourth | JKN - contributory | 21% | 28% | -5·48 (-7·51 – -3·45)** | -4·21 (-6·33 – -2·08)** |
|  | JKN - subsidised | 45% | 35% | 4·89 (2·69 – 7·09)** | 4·63 (2·43 – 6·82)** |
|  | Other insurance | 5% | 9% | -2·41 (-3·56 – -1·25)** | -2·33 (-3·50 – -1·17)** |
|  | No insurance | 29% | 28% | 2·99 (0·70 – 5·28)* | 1·91 (-0·34 – 4·16) |
| Highest | JKN - contributory | 43% | 48% | -5·70 (-8·30 – -3·10)** | -3·67 (-6·19 – -1·15)** |
|  | JKN - subsidised | 31% | 21% | 5·28 (3·14 – 7·42)** | 4·58 (2·42 – 6·74)** |
|  | Other insurance | 7% | 10% | -0·94 (-2·84 – 0·97) | -0·96 (-2·90 – 0·98) |
|  | No insurance | 19% | 21% | 1·35 (-0·85 – 3·56) | 0·05 (-2·18 – 2·28) |

Supplementary 4. Factors Associated with Health Insurance Enrolment amongst People with Disabilities

| Variables | No insurance  AME – PP (95% CI) | JKN  AME – PP (95% CI) | Multiple & other insurance  AME – PP (95% CI) | Subsidised (vs contributory)  AME – PP (95% CI) |
| --- | --- | --- | --- | --- |
| Sex |  |  |  |  |
| Male | Ref | Ref | Ref | Ref |
| Female | 0·17 (-1·30 – 1·66) | -1·23 (-2·82 – 0·32) | 1·05 (0·01 – 2·09) | 0·56 (-1·20 – 2·31) |
| Age |  |  |  |  |
| <18 | Ref | Ref | Ref | Ref |
| 18-30 | -9·67 (-14·25 – -5·09)** | 11·93 (7·22 – 16·63)** | -2·26 (-5·29 – 0·77) | 9·71 (4·35 – 15·07)** |
| 31-40 | -6·94 (-11·62 – -2·26)** | 9·33 (4·54 – 14·12)** | -2·39 (-5·31 – 0·53) | 7·82 (2·27 – 13·38)** |
| 41-50 | -9·63 (-14·05 – -5·21)** | 13·09 (8·69 – 17·49)** | -3·46 (-6·10 – -0·83)* | 10·53 (5·61 – 15·46)** |
| 51-60 | -14·08 (-18·05 – -10·11)** | 18·29 (14·30 – 22·28)** | -4·21 (-6·76 – -1·67)** | 5·89 (1·16 – 10·62)* |
| >60 | -9·66 (-13·36 – -5·95)** | 13·60 (9·94 – 17·25)** | -3·94 (-13·36 – -5·95)** | 9·17 (4·88 – 13·47)** |
| Residence |  |  |  |  |
| Urban | Ref | Ref | Ref | Ref |
| Rural | 9·45 (7·52 – 11·37)** | -8·05 (-10·03 – -6·06)** | -1·40 (-2·69 – -0·10)* | 19·20 (16·99 – 21·41)** |
| Household size |  |  |  |  |
| 1-2 members | Ref | Ref | Ref | Ref |
| 3-4 members | -5·73 (-8·06 – -3·40)** | 5·92 (3·56 – 8·28)** | -0·19 (-1·68 – 1·29) | -1·43 (-4·12 – 1·26) |
| 5+ members | -5·79 (-8·20 – -3·38)** | 5·49 (3·04 – 7·94)** | 0·30 (-1·31 – 1·91) | 0·06 (-2·69 – 2·82) |
| Education |  |  |  |  |
| No school/less than elementary | Ref | Ref | Ref | Ref |
| Elementary | -4·87 (-7·11 – -2·63)** | 4·61 (2·30 – 6·93)** | 0·26 (-1·23 – 1·75) | -10·05 (-12·27 - -7·83)** |
| Junior high school | -6·27 (-9·41 – -3·12)** | 7·30 (4·06 – 10·53)** | -1·03 (-2·89 – 0·83) | -20·89 (-24·12 - -17·67)** |
| Senior high school | -13·49 (-16·51 – -10·48)** | 9·24 (5·89 – 12·59)** | 4·25 (1·81 – 6·69)** | -39·47 (-42·78 - -36·16)** |
| College/university | -16·60 (-20·46 – -12·74)** | 10·83 (6·07 – 15·58)** | 5·77 (1·58 – 9·96)** | -46·11 (-50·77 - -41·46)** |
| Marital |  |  |  |  |
| Never married | Ref | Ref | Ref | Ref |
| Married | -7·55 (-11·06 – -4·04)** | 5·85 (2·18 – 9·48)** | 1·72 (-0·64 – 4·08) | -15·67 (-20·04 - -11·30)** |
| Widowed/divorced | -0·74 (-4·77 – 3·29) | -0·89 (-4·96 – 3·17) | -1·63 (-1·01 – 4·28) | -8·88 (-13·57 – 4·19)** |
| Working status |  |  |  |  |
| Not working | Ref | Ref | Ref | Ref |
| Self-employed | 1·96 (-0·35 – 4·26) | -2·09 (-4·48 – 0·30) | -0·13 (-1·31 – 1·57) | 3·90 (1·46 – 6·34)** |
| Working for other | -5·10 (-8·80 – -1·40)** | 0·49 (-3·71 – 4·68) | 4·61 (1·45 – 7·77)** | -11·37 (-16·12 - -6·62)** |
| Expenditure per capita quintiles |  |  |  |  |
| Lowest | Ref | Ref | Ref | Ref |
| Second | -4·31 (-7·17 – -1·46)** | 3·74 (0·88 – 6·61)* | 0·57 (-1·15 – 2·29) | -9·11 (-11·68 - -6·54)** |
| Third | -7·04 (-9·93 – -4·14)** | 7·34 (4·42 – 10·26)** | -0·30 (-1·97 – 1·36) | -17·41 (-20·36 - -14·46)** |
| Fourth | -7·53 (-10·59 – -4·46)** | 6·81 (3·74 – 9·88)** | 0·72 (-1·09 – 2·52) | -28·87 (-32·15 - -25·60)** |
| Highest | -16·66 (-19·77 – -13·54)** | 9·99 (6·45 – 13·54)** | 6·66 (3·94 – 9·39)** | -53·19 (-56·65 - -49·74)** |

Abbreviation: AME: Average Marginal Effects, PP: percentage points, CI: Confidence Interval

AME – controlled for age, sex, urban/rural, and household size

*P<0·05; **P<0·01

Supplementary 5. Healthcare Utilization and Financial Protection between People with versus without Disability by Insurance Status in Indonesia

|  | **Overall**  AME – PP (95% CI) | **No insurance**  AME – PP (95% CI) | **JKN only**  AME – PP (95% CI) | **Among JKN-subsidised**  AME – PP (95% CI) | **Among JKN-contributory**  AOR (95% CI) | **Multiple/other insurance**  AOR (95% CI) |
| --- | --- | --- | --- | --- | --- | --- |
| Having outpatient visits in the last month | 8·01 (7·30 – 8·72)** | 5·46 (4·17 – 6·75)** | 9·14 (8·24 – 10·03)** | 7·73 (6·76 – 8·71)** | 13·46 (11·57 – 15·35)** | 8·76 (6·64 – 10·88)** |
| Having > 1 outpatient visits^#^ | 9·61 (7·99 – 11·24)** | 7·38 (4·33 – 10·44)** | 9·87 (7·95 – 11·80)** | 9·37 (7·22 – 11·52)** | 10·81 (7·21 – 14·41)** | 9·12 (4·05 – 14·20)** |
| Having inpatient visits in the last year | 3·74 (3·32 – 4·16)** | 1·03 (0·56 – 1·51)** | 5·08 (4·48 – 5·69)** | 4·09 (3·48 – 4·69)** | 8·36 (6·89 – 9·83)** | 3·67 (2·26 – 5·07)** |
| Hospital length of stay ≥ 3 days^#^ | 9·47 (6·82 – 12·12)** | 10·61 (2·46 – 18·76)* | 8·24 (5·33 – 11·15)** | 7·43 (4·07 – 10·78)** | 10·01 (5·80 – 14·22)** | 9·52 (3·48 – 15·56)** |
| Out-of-pocket payment |  |  |  |  |  |  |
| Lowest | -1·87 (-2·84 – -0·89)** | 0·06 (-1·91 – 2·03) | -3·05 (-4·19 – -1·90)** | -4·60 (-5·92 – -3·27)** | -4·50 (-6·40 – -2·59)** | -1·30 (-3·99 – 1·39) |
| Second | -3·50 (-4·44 – -2·56)** | -4·00 (-5·85 – -2·16)** | -3·36 (-4·51 – -2·21)** | -3·04 (-4·34 – -1·74)** | -5·96 (-8·13 – -3·80) | -2·71 (-5·72 – 3·01) |
| Highest | 5·37 (4·35 – 6·39)** | 3·94 (2·13 – 5·76)** | 6·41 (5·13 – 7·68)** | 7·64 (6·25 – 9·02)** | 10·46 (8·00 – 12·92)** | 4·01 (0·87 – 7·15)* |
| Experiencing Catastrophic Health Expenditure (40% threshold) | 1·17 (0·91 – 1·43)** | 0·59 (0·28 – 0·91)** | 1·37 (1·01 – 1·72)** | 1·16 (0·77 – 1·56)** | 2·68 (1·78 – 3·59)** | 1·74 (0·56 – 2·93)* |

Abbreviation: AME: Average Marginal Effects, PP: percentage points, CI: Confidence Interval

AME – controlled for age, sex, urban/rural, and household size

*P<0·05, **P<0·01

^#^ Among those using the services

Supplementary 6. Association between Health Insurance and Healthcare Utilization, and Financial Protection by Insurance Status among People with Disabilities in Indonesia

| Outcome variables | Predictor variables - AME – PP (95% CI) | | |
| --- | --- | --- | --- |
|  | JKN vs no insurance | JKN vs Multiple/other insurance | JKN Subsidised vs contributory |
| Having outpatient visits in the last month | 6·56 (5·12 – 8·01)** | 1·09 (-1·16 – 3·35) | -6·38 (-8·51 – -4·25)** |
| Having > 1 outpatient visits^#^ | 4·12 (0·41 – 7·84)* | 1·39 (-4·16 – 6·93) | 2·12 (-2·22 – 6·50) |
| Having inpatient visits in the last year | 6·19 (5·33 – 7·05)** | 1·93 (0·48 – 3·38** | -6·35 (-8·01 – -4·70)** |
| Hospital length of stay ≥ 3 days^#^ | 10·11 (2·38 – 17·85)* | 0·21 (-5·91 – 6·34) | -5·58 (-9·88 – -1·28)* |
| Out-of-pocket payment |  |  |  |
| Lowest | -10·92 (-13·17 – -8·66)** | -3·96 (-7·89 - -0·03)* | 20·91 (18·52 – 23·31)** |
| Second | -2·01 (-4·13 – 0·11) | -3·24 (-7·31 – 0·83) | 6·18 (3·69 – 8·67)** |
| Highest | 12·93 (10·81 – 15·04)** | 7·20 (3·01 – 11·39)** | -27·09 (-29·90 – -24·29)** |
| Experiencing Catastrophic Health Expenditure (40% threshold) | 1·18 (0·67 – 1·69)** | -0·31 (-1·57 – 0·95) | -2·10 (-3·17 – -1·03)** |

Abbreviation: AME: Average Marginal Effects, PP: percentage points, CI: Confidence Interval

AME – controlled for age, sex, urban/rural, and household size

*P<0·05, **P<0·01

^#^ Among those using the services

Supplementary 7. Interaction between age and sex in the association between disability status and health insurance coverage

| **Variable** | **Reference category** | **JKN only vs no insurance**  **AOR (95% CI)** | **Other/Multiple Insurance vs no insurance**  **AOR (95% CI)** |
| --- | --- | --- | --- |
| Age group x sex | Males aged 0-17 years |  |  |
| 18–30 years × Female |  | 1·08 (1·05–1·12)** | 1·06 (1·01–1·11)* |
| 31–40 years × Female |  | 1·14 (1·10–1·17)** | 1·05 (1·00–1·10)* |
| 41–50 years × Female |  | 1·12 (1·08–1·16)** | 1·02 (0·97–1·07) |
| 51–60 years × Female |  | 1·09 (1·05–1·13)** | 1·00 (0·95–1·06) |
| >60 years × Female |  | 1·00 (0·96–1·04) | 0·95 (0·90–1·01) |
| Among people with disabilities Age group x sex | Males aged 0-17 years |  |  |
| 18–30 years × Female |  | 1·32 (0·89 – 1·98) | 1·18 (0·65 – 2·15) |
| 31–40 years × Female |  | 1·27 (0·83 – 1·95) | 1·30 (0·71 – 2·38) |
| 41–50 years × Female |  | 1·07 (0·74 – 1·56) | 0·51 (0·29 – 0·88)* |
| 51–60 years × Female |  | 1·07 (0·77 – 1·50) | 0·84 (0·50 – 1·41) |
| >60 years × Female |  | 0·89 (0·67 – 1·19) | 0·78 (0·50 – 1·20) |
| Disability x age group x sex | Persons without disability, males aged 0–17 years |  |  |
| Yes × 18–30 years × Female |  | 1·23 (0·82–1·83) | 1·11 (0·61–2·02) |
| Yes × 31–40 years × Female |  | 1·12 (0·74–1·72) | 1·23 (0·67–2·27) |
| Yes × 41–50 years × Female |  | 0·96 (0·66–1·40) | 0·50 (0·28–0·87)* |
| Yes × 51–60 years × Female |  | 1·00 (0·71–1·40) | 0·84 (0·50–1·41) |
| Yes × >60 years × Female |  | 0·90 (0·67–1·20) | 0·81 (0·52–1·26) |
